# Rifampin-warfarin interaction in a mitral valve replacement patient receiving rifampin for infective endocarditis: a case report

**DOI:** 10.1186/s40064-015-1653-8

**Published:** 2016-01-04

**Authors:** Amr Mohamed Fahmi, Osama Abdelsamad, Hazem Elewa

**Affiliations:** Clinical Pharmacist, Pharmacy department, Alwakra Hospital, P.O. Box: 82228, Doha, Qatar; Clinical Pharmacy and Practice Section, College of Pharmacy, Qatar University, P.O. Box 2713, Doha, Qatar

**Keywords:** Warfarin, Rifampin, Mitral valve replacement, Infective endocarditis

## Abstract

**Introduction:**

Warfarin therapy is associated with many drug interactions that may cause a significant alteration in its anticoagulant effect. Rifampin is a widely used antimicrobial that has major interactions with several medications including warfarin due to its strong P-glycoprotein and liver enzyme inducer activity especially on CYP2C9, CYP3A4, CYP1A2 and CYP2C19.

**Presentation:**

We report a case of a 34-year-old Srilankan female chronically treated with warfarin for her mitral valve replacement. The patient developed infective endocarditis and was started on a 6-week treatment with rifampin along with other antibiotics. Warfarin dose was increased from 52.5 to 210 mg/week over the course of the rifampin therapy, however, the INR remained subtherapeutic throughout the whole period and reached 2.4 by the end of rifampin therapy.

**Discussion and evaluation:**

Anticoagulation management was challenging in the period following the end of rifampin therapy as well, and multiple dose adjustments starting with an increase and followed by reduction were required till she was stable on an 80 mg/week warfarin dose 5 weeks post-rifampin therapy.

**Conclusion:**

Our findings suggest the importance of close monitoring of warfarin therapy during and after the use of rifampin to minimize the risks of under and over-anticoagulation and improve the safety and efficacy of warfarin therapy.

## Introduction

For over 60 years, warfarin has been the mainstay anticoagulant used in the prevention and treatment of thromboembolic complications in patients with atrial fibrillation, venous thromboembolism, prosthetic heart valves, and coronary artery disease (Nutescu et al. 2006). Because of its narrow therapeutic index and the substantial interpatient variability associated with its dosing, careful monitoring of warfarin is strongly recommended by current practice guidelines to minimize the risks associated with warfarin’s inadequate dosing and ensure optimal outcomes for anticoagulated patients (Horton and Bushwick 1999; You et al. 2012). Despite its wide-spread use, management of patients on warfarin treatment is associated with many challenges. These challenges include therapeutic dose inter- and intra-patient variability, drug and food interactions, environmental factors, co-morbidities, and narrow therapeutic index (Nutescu et al. 2006). Accordingly, close monitoring and individualized dosing based on International normalized ratio (INR) measurement is warranted to confirm effective level of anticoagulation (Ageno et al. 2012).

Warfarin is administered as a racemic mixture, the R and S enantiomers. The more potent, S enantiomer, is subject to approximately 90 % oxidative metabolism, mainly through the Cytochrome P450 2C9 (CYP2C9) enzyme of the Cytochrome P450 system and to a minor extent by CYP3A4. On the other hand, the less potent, R enantiomer, is subject to approximately 60 % oxidative metabolism, primarily through CYP1A2 and CYP3A4, and to a minor extent through CYP2C19 (Kaminsky and Zhang 1997; Miners and Birkett 1998; Cropp and Bussey 1997).

Rifampin is a widely used antimicrobial for the treatment of challenging and life-threatening infections such as tuberculosis, meningitis, osteomyelitis and endocarditis (Baciewicz et al. 2008). Due to its proven efficacy in the management of infective endocarditis, American Heart association (AHA), and the European Society for cardiology (ESC),recommend rifampin in treatment of prosthetic valve endocarditis caused by Coagulase-Negative Staphylococci species in combination with other antimicrobial agents (Gould et al. 2012; Newburger et al. 2004). Rifampin is known to have major interactions with several medications due to its strong P-glycoprotein and liver enzyme inducer activity especially on CYP2C9, CYP3A4, CYP1A2 and CYP2C19 (Horn et al. 2007; Ohno et al. 2008). Rifampin’s concomitant use with warfarin results in a clinically significant drug–drug interaction. This interaction leads to accelerated warfarin’s clearance and ultimately a reduction in its anticoagulant effect (Strayhorn et al. 1997; Baciewicz et al. 2008). There are several, well-documented cases of potential interactions during concurrent rifampin-warfarin use (Lee and Thrasher 2001; Martins et al. 2013; Krajewski 2010; Maina et al. 2013). This interaction affects warfarin dose required to achieve and maintain therapeutic INR and may increase this dose up to six times to reach target therapeutic INR. If warfarin dose is not adjusted accordingly, this may lead to subtherapeutic INR values and increase in the risk of clinical complications (Kim et al. 2007; Tong et al. 2014; Krajewski 2010; Lee and Thrasher 2001). This interaction remains clinically significant during, and weeks after discontinuation of rifampin. Additionally, risk of bleeding after rifampin discontinuation has been reported (Martins et al. 2013).

The aim of this case report is to address the effect of rifampin–warfarin interaction in a patient with endocarditis who has been receiving a stable and therapeutic warfarin dose for mechanical heart valve replacement. The patient has provided consent for the use of his personal and medical information in this case report.

## Presentation

A 34-year-old Srilankan female with a history of rheumatic heart disease, underwent mitral valve replacement in 1994 and was on anticoagulation ever since with warfarin and a target INR of (2.5–3.5). She was maintained on warfarin weekly dose of 52.5 mg (7.5 mg daily) since she started follow-up at our pharmacist-managed anticoagulation clinic in late 2013.

On the 23rd of October, 2014, the patient started to have fever with chills and was admitted to the hospital and diagnosed with prosthetic valve infective endocarditis. Initially, the patient was started on vancomycin (1800 mg/day), gentamicin (80 mg/day), ceftriaxone (2 g/day) and rifampin (600 mg/day). The patient was using her usual warfarin dose (7.5 mg daily) and her INR was 2.6. Two days later, and she complained of heavy menses and her hemoglobin dropped to 7.5 g/dl. INR increased to 4.4 at that time and warfarin was stopped.

Three days later, some changes were made to her antibiotic regimen where vancomycin was replaced by daptomycin (300 mg/day). Her gentamycin was stopped after 3 weeks from its initiation while the rest of the antibiotics (daptomycin, ceftriaxone and rifampin) were continued for three more weeks to complete a six-week course.

After being held for 5 days due to the bleeding and drop in hemoglobin, warfarin was restarted and an INR of 1.6 was recorded at that time. Since warfarin resumption, the patient’s INR became subtherapeutic likely due to the interaction with rifampin. Conservative increments of warfarin dose were made during her hospitalization period which continued for almost a month. Upon discharge on the 18th of November, INR was 1.3 and warfarin weekly dose was 105 mg (15 mg/day), she was then followed-up again at the anticoagulation clinic and was bridged with therapeutic dose enoxaparin (1 mg/kg subcutaneous every 12 h). Warfarin weekly dose was increased gradually from 105 to 155 mg over the following 2 weeks but her INR remained subtherapeutic at all time and increased slightly from 1.3–1.8.

**Fig. 1:**
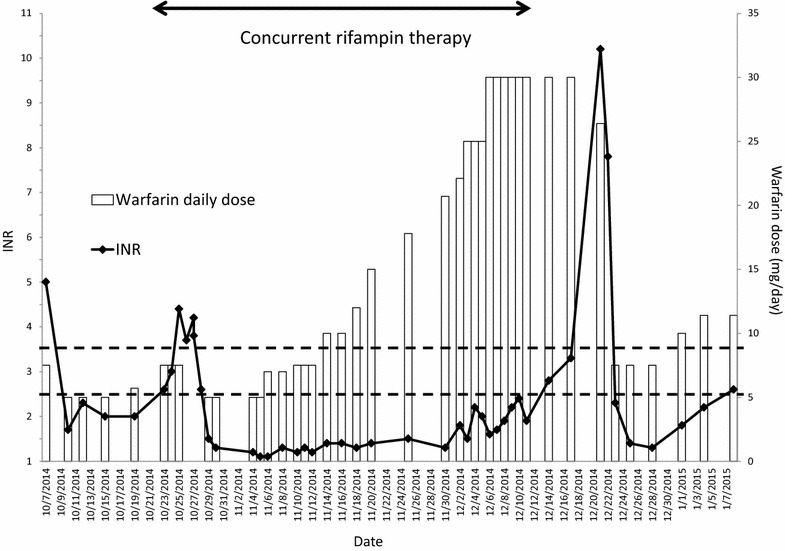
This *graph* represents the daily warfarin dose, INR, and concomitant rifampin therapy over time. The *x-axis* represents time when the INR was measured. The left *y-axis* represents the INR units and is shown by the *black diamond points*. The right *Y-axis* represents warfarin dose administered in milligrams/day and is shown by the *vertical bars*. The therapeutic range is indicated between the *two* *dotted lines* (2.5–3.5). The time period of concomitant rifampin administration is shown as the horizontal solid 2-way arrow

On the 3rd of December, the patient had symptoms of palpitations and her electrocardiogram showed atrial flutter with rapid ventricular response of 147 beats per minute and she was admitted again to the cardiac care unit for rate control were she was maintained on metoprolol (50 mg/day). Six days later, the patient’s antibiotics including rifampin were stopped after completing a total of 6-week treatment course for endocarditis. The following day, she was discharged with an INR of 2.4 and a warfarin weekly dose of 210 mg daily (30 mg/day). The first significant change in her INR was a drop to 1.9 (no change in dosing was made at this point) and she was followed-up every 2–3 days. Her INR readings gradually increased but were all in therapeutic range (INR on 17/12/2014 was 3.3). Four days later, (11 days post-antibiotics discontinuation, the patient had a supratherapeutic INR of 10.2 for which warfarin was held for 2 days and then resumed at a reduced-dose of 7.5 mg/day (her routine dose prior to rifampin initiation). Patient’s INR did not adequately respond to this dose reduction and became subtherapeutic. Warfarin dose was increased gradually while bridging with therapeutic dose enoxaparin till she reached a maintenance dose of (10 mg daily except 15 mg twice/week) (a weekly dose of 80 mg) on 15/1/2015 where her INR reached 2.5 (Fig. 1).

In summary, warfarin dose was increased from 52.5 to 210 mg/week over the course of the 6-week treatment with antibiotics for her infective endocarditis. Due to the patient’s risk of stroke and the difficulty to reach therapeutic INR, she was bridged using enoxaparin. The patient’s INR remained subtherapeutic throughout the whole period when she received rifampin concomitantly with warfarin, and reached 2.4 by the end of rifampin therapy. Anticoagulation management was challenging in the period following the end of her rifampin therapy, and multiple dose adjustments starting with an increase and followed by gradual reduction were required till she was stable on an 80 mg/week warfarin dose 5 weeks post-rifampin therapy.

## Discussion

In this case report, we observed the challenging management of warfarin therapy when rifampin therapy along with other antibiotics (ceftriaxone, gentamicin and daptomycin) were used concomitantly in a patient previously stable on anticoagulation therapy with warfarin for stroke prevention from her mechanical mitral valve replacement. While on rifampin, she required about fourfold increase in her warfarin dose and the addition of bridging therapy using low molecular weight heparin (LMWH) to maintain proper anticoagulation due to failure in achieving therapeutic INR with warfarin. After discontinuation of rifampin, the drug–drug interaction effect remained for almost 5 weeks. After the offset of rifampin effect, warfarin maintenance dose was noticed to be 150 % higher than its baseline prior to rifampin initiation (80 vs 52.5 mg/week). It is also important to note that the sudden elevation and drop in INR associated with the initiation and discontinuation of the antibiotic treatment, respectively is likely mediated by the interaction between warfarin and ceftriaxone. A previous report has shown that concomitant use of ceftriaxone and warfarin can lead to a significant elevation in INR and warfarin anticoagulant effect (Clark and Burns 2011).

Several reports have previously described the interaction between rifampin and warfarin and the effect of rifampin on the anticoagulation effect of warfarin during concomitant use of both medications (Lee and Thrasher 2001; Martins et al. 2013; Krajewski 2010; Maina et al. 2013). Lee et al. described a case for a patient who was started on rifampin for active tuberculosis and was started on warfarin 4 months later for left ventricular thrombus. The warfarin dosage required to attain therapeutic INR was significantly higher when the patient received concomitant rifampin (233 % higher) than the dose required after rifampin discontinuation (Lee and Thrasher 2001). Similar reports were described by others (Self and Mann 1975; Almog et al. 1988).

Additional case studies described patients who were started on rifampin 600 mg and warfarin around the same time. The warfarin dose was reduced by about 50 % to maintain therapeutic INR after discontinuation of rifampin (Krajewski 2010; Romankiewicz and Ehrman 1975).

Other reports have described the interaction between warfarin and rifampin in patients already on chronic warfarin therapy (Martins et al. 2013; Tong et al. 2014; Kim et al. 2007). Similar to our case report, these reports described the interaction resulting from the addition of rifampin in patients already receiving chronic warfarin therapy. Sequential increase in warfarin dose was required to reach therapeutic level with frequent INR monitoring. After discontinuation of rifampin, the warfarin dosage requirement was reduced gradually to avoid supratherapeutic INR until reaching therapeutic level.

Collectively, all previously described case studies and case series agree on the difficulty of achieving therapeutic level of anticoagulation despite escalation of warfarin dose due to the interaction with rifampin. The increments recorded in warfarin dose vary between twofold to sixfold compared to the dose required when patient is not on rifampin. The onset of interaction and the requirement to increase warfarin dose seem to have faster timeline (7–14 days) compared to the offset of the interaction which may require tapering the warfarin dose down over weeks. There are wide inter-patient variability in the extent, onset and offset of the warfarin-rifampin interaction.

## Conclusion

This case study confirms previously reported studies on the interaction between warfarin and rifampin. It also demonstrates that this interaction is clinically significant and can be very critical in high-risk patients such as those receiving warfarin for mechanical valve replacement. Our findings suggest the importance of close monitoring of warfarin therapy during and after the use of rifampin to minimize the risks of under and over-anticoagulation and improve the safety and efficacy of warfarin therapy.

## Abbreviations

INR: International normalized ratio
AHA: American heart association
ESC: European Society for cardiology
LMWH: low molecular weight heparin

## References

[CR1] Ageno W, Gallus AS, Wittkowsky A, Crowther M, Hylek EM, Palareti G (2012). Oral anticoagulant therapy: antithrombotic therapy and prevention of thrombosis, 9th ed: American College of chest physicians evidence-based clinical practice guidelines. Chest.

[CR2] Almog S, Martinowitz U, Halkin H, Bank HZ, Farfel Z (1988). Complex interaction of rifampin and warfarin. South Med J.

[CR3] Baciewicz AM, Chrisman CR, Finch CK, Self TH (2008). Update on rifampin and rifabutin drug interactions. Am J Med Sci.

[CR4] Clark TR, Burns S (2011). Elevated international normalized ratio values associated with concomitant use of warfarin and ceftriaxone. Am J Health Syst Pharm.

[CR5] Cropp JS, Bussey HI (1997). A review of enzyme induction of warfarin metabolism with recommendations for patient management. Pharmacotherapy.

[CR6] Gould FK, Denning DW, Elliott TS, Foweraker J, Perry JD, Prendergast BD, Sandoe JA, Spry MJ, Watkin RW, Working Party of the British Society for Antimicrobial C (2012). Guidelines for the diagnosis and antibiotic treatment of endocarditis in adults: a report of the Working Party of the British Society for Antimicrobial Chemotherapy. J Antimicrob Chemother.

[CR7] Horn JR, Hansten PD, Chan LN (2007). Proposal for a new tool to evaluate drug interaction cases. Ann Pharmacother.

[CR8] Horton JD, Bushwick BM (1999). Warfarin therapy: evolving strategies in anticoagulation. Am Fam Physician.

[CR9] Kaminsky LS, Zhang Z-Y (1997). Human P450 metabolism of warfarin. Pharmacol Ther.

[CR10] Kim KY, Epplen K, Foruhari F, Alexandropoulos H (2007). Update on the interaction of rifampin and warfarin. Prog Cardiovasc Nurs.

[CR11] Krajewski KC (2010). Inability to achieve a therapeutic INR value while on concurrent warfarin and rifampin. J Clin Pharmacol.

[CR12] Lee CR, Thrasher KA (2001). Difficulties in anticoagulation management during coadministration of warfarin and rifampin. Pharmacotherapy.

[CR13] Maina MW, Pastakia SD, Manji I, Kirui N, Kirwa C, Karwa R (2013). Describing the profile of patients on concurrent rifampin and warfarin therapy in western Kenya: a case series. Drugs R D.

[CR14] Martins MA, Reis AM, Sales MF, Nobre V, Ribeiro DD, Rocha MO, Ribeiro AL (2013). Rifampicin-warfarin interaction leading to macroscopic hematuria: a case report and review of the literature. BMC Pharmacol Toxicol.

[CR15] Miners JO, Birkett DJ (1998). Cytochrome P4502C9: an enzyme of major importance in human drug metabolism. Br J Clin Pharmacol.

[CR16] Newburger JW, Takahashi M, Gerber MA, Gewitz MH, Tani LY, Burns JC, Shulman ST, Bolger AF, Ferrieri P, Baltimore RS, Wilson WR, Baddour LM, Levison ME, Pallasch TJ, Falace DA, Taubert KA (2004). Diagnosis, treatment, and long-term management of Kawasaki disease: a statement for health professionals from the Committee on Rheumatic Fever, Endocarditis and Kawasaki Disease, Council on Cardiovascular Disease in the Young American Heart Association. Circulation.

[CR17] Nutescu EA, Shapiro NL, Ibrahim S, West P (2006). Warfarin and its interactions with foods, herbs and other dietary supplements. Expert Opin Drug Saf.

[CR18] Ohno Y, Hisaka A, Ueno M, Suzuki H (2008). General framework for the prediction of oral drug interactions caused by CYP3A4 induction from in vivo information. Clin Pharmacokinet.

[CR19] Romankiewicz JA, Ehrman M (1975). Rifampin and warfarin: a drug interaction. Ann Intern Med.

[CR20] Self TH, Mann RB (1975). Interaction of rifampin and warfarin. Chest.

[CR21] Strayhorn VA, Baciewicz AM, Self TH (1997). Update on rifampin drug interactions, III. Arch Intern Med.

[CR22] Tong EY, Kowalski M, Yip GS, Dooley MJ (2014). Impact of drug interactions when medications are stopped: the often forgotten risks. Med J Aust.

[CR23] You JJ, Singer DE, Howard PA, Lane DA, Eckman MH, Fang MC, Hylek EM, Schulman S, Go AS, Hughes M, Spencer FA, Manning WJ, Halperin JL, Lip GY, American College of Chest Physicians (2012). Antithrombotic therapy for atrial fibrillation: antithrombotic Therapy and Prevention of Thrombosis, 9th ed: American College of Chest Physicians Evidence-Based Clinical Practice Guidelines. Chest.

